# TME-activatable theranostic nanoplatform with ATP burning capability for tumor sensitization and synergistic therapy

**DOI:** 10.7150/thno.44569

**Published:** 2020-05-25

**Authors:** Yuanli Luo, Bin Qiao, Ping Zhang, Chao Yang, Jin Cao, Xun Yuan, Haitao Ran, Zhigang Wang, Lan Hao, Yang Cao, Jianli Ren, Zhiyi Zhou

**Affiliations:** 1 Ultrasound Department of the Second Affiliated Hospital of Chongqing Medical University, Chongqing Key Laboratory of Ultrasound Molecular Imaging, Chongqing, 400010, P. R. China.; 2 Radiology Department of Chongqing General Hospital, University of Chinese Academy of Sciences, Chongqing 400014, P. R. China.; 3 Ophthalmology Department of the Second Affiliated Hospital of Chongqing Medical University, Chongqing, 400010, P. R. China.; 4 General Practice Department of Chongqing General Hospital, University of Chinese Academy of Sciences, Chongqing 400014, P. R. China.

**Keywords:** adenosine triphosphate, responsive drug release, anti-tumor therapy, chemosensitivity, synergistic therapy, multimodal imaging

## Abstract

Adenosine triphosphate (ATP), as a key substance for regulating tumor progression in the tumor microenvironemnt (TME), is an emerging target for tumor theranostics. Herein, we report a minimalist but versatile nanoplatform with simultaneously TME-responsive drug release, TME-enhanced imaging, ATP-depletion sensitized chemotherapy and photothermal therapy for intelligent tumor theranostics.

**Methods:** The Fe^3+^ and tannic acid (TA) coordination were self-deposited on doxorubicin (Dox) in a facile method to prepare Dox-encapsulated nanoparticles (DFTNPs).

**Results:** When irradiated by a near infrared laser, the DFTNPs could elevate the temperature in the tumor region efficiently. Subsequently, the Dox could be released by the disassembly of Fe^3+^/TA in the TME to initiate chemotherapy. Particularly, the smart nanoagent not only enabled ATP-depletion and enhanced the therapeutic effect of chemotherapy, but also acted as photothermal transduction agent for photothermal therapy. Moreover, the nanoagent also acted as T_1_-weighted MR imaging,photoacoustic imaging and photothermal imaging contrast agent. The mice treated by DFTNPs plus laser showed a complete tumor eradication in 14d observation.

**Conclusion:** This as-prepared versatile nanoplatform offers new insights toward the application of smart nanoagents for improved tumor theranostics.

## Introduction

Chemotherapy has been regarded as one of the most effective anticancer modalities in the clinic, which utilizes chemotherapeutic drugs to interfere with the process of tumor cell division and proliferation and subsequently induce tumor cell death 1-3. However, the therapeutic efficacy is largely limited by the lack of chemosensitivity of tumor cells to the chemotherapeutic drug, which can lead to 90% of cancer recurrence 4. To deal with this challenge, photothermal therapy (PTT) is carried out, which can convert light energy into heat for irreversible tumor damage with the advantage of high spatiotemporal selectivity, excellent tumor ablation efficacy and minimal systemic toxicity 5-7. Besides, it has been well documented that the combining of PTT with chemotherapy may result in remarkable superadditive effects (namely “1+1>2”), which is much stronger than single modality therapy or their theoretical combination 8. However, the light penetration during PTT is always limited, which will substantially hinder the synergistic therapeutic efficiency due to an intrinsic lack of chemosensitivity 9-11.

Currently, significant efforts have been exploited to increase the chemosensitivity, including combination therapy with gene, gas molecules, small molecules inhibitors and reactive oxygen species (ROS) generating systems 12-14. However, most of these approaches might encounter various challenges toward clinical applications due to the incorporation of cytotoxic molecules. Moreover, non-specific drug release during circulation poses a serious threat on the biosafety for *in vivo* applications 8. Fortunately, the emergence of stimuli-responsive nanomedicine, a promising strategy for drug delivery with reduced systemic toxicity, can provide an ideal choice for on-demand drug release under certain stimuli in the tumor microenvironment (TME) 15-18. Therefore, it is a tendency to explore an efficient on-demand drug release strategy that is capable of enhancing chemosensitivity and achieving PTT. ATP, as one of the important abnormal substances that provide energy for tumor progression, is highly upregulated in tumor cells (1-10×10^-3^ M) mainly due to the excessive glycolysis and has become an emerging target for tumor therapy 19-22. Importantly, ATP-dependent drug efflux significantly decreases drug accumulation and efficacy in the therapeutic process 17,23. Accordingly, depleting intracellular ATP contents can effectively increase cancer cell chemosensitivity and hence, enhance the therapeutic efficacy of chemotherapeutic drugs. Moreover, acid extracellular microenvironment (pH 6.5-6.8) and endosome/lysosome microenvironment (pH 4.5-5.5), caused by the excessive secretion of lactate and other tumor cell metabolites, can be exploited to induce the crush of nanoparticles in a spatially controlled manner and subsequently trigger on-demand drug release. As far as we know, it is rarely reported to improve the sensitivity of chemotherapy by depleting intracellular ATP for sensitizing tumor cells with pH/ATP responsive drug release properties.

The rational integration of multimodal imaging, on-demand drug release, and photothermal therapy into a versatile oncotherapy nanoplatform has shown great potential in cancer theranostics 24-28. Multimodal imaging-guided nanosized drug delivery systems (NDDS) can provide essential information regarding solid tumors and show high potential in guiding the therapeutic process, evaluating the therapeutic effect and trimming the therapeutic time window in real-time 29-32. Recently, ferric ion (Fe^3+^)-crosslinked tannic acid (TA) as a multifunctional carrier has emerged and shown broad prospects for achieving synergistic therapy 33-38. Fe^3+^/TA with multiple imaging capabilities is an intriguing candidate potentially serving as an efficient imaging contrast. Generally, it would be paramount to achieve the real-time track of the nanoagents after systemic administration for further guiding the therapeutic process and monitoring the therapeutic response 39,40. Among various imaging paradigms, magnetic resonance imaging (MRI) is a widely used technique for cancer diagnosis with high spatial resolution and penetration depth, but its sensitivity and spatial resolution remain unsatisfactory for detecting nanoagents 24,41,42. Considering the protonation of hydroxyl groups at low pH, the Fe^3+^/TA tends to destabilize and disassemble rapidly, which leads to the disassembly of the cross-links 37. Moreover, ATP has shown a strong binding ability to various metal ions through metal ion-triphosphate coordination, which may compete with TA and corrode Fe^3+^/TA for the release of encapsulated drugs. In our report, Fe^3+^/TA was used as host materials for pH/ATP responsive drug release and serves as a pH/ATP sensor for “turn-on” MRI imaging capability due to the strong binding affinity of Fe^3+^ to ATP. Moreover, Fe^3+^/TA can serve as photoacoustic imaging (PAI) and photothermal imaging (PTI) contrast agents. PAI could permit not only high contrast and resolution images due to its optical absorption and ultrasonic detection properties but also functional information of biological tissues and organizational distribution information of PAI contrast 40,42,43. Besides, PTI could provide real-time thermal dynamic information in the PTT process. Therefore, it is highly desired to integrate PAI, MRI and PTI into one single nanoagent to provide complementary information to compensate each other, which may facilitate the guidance of the whole therapeutic process.

**Scheme 1:**
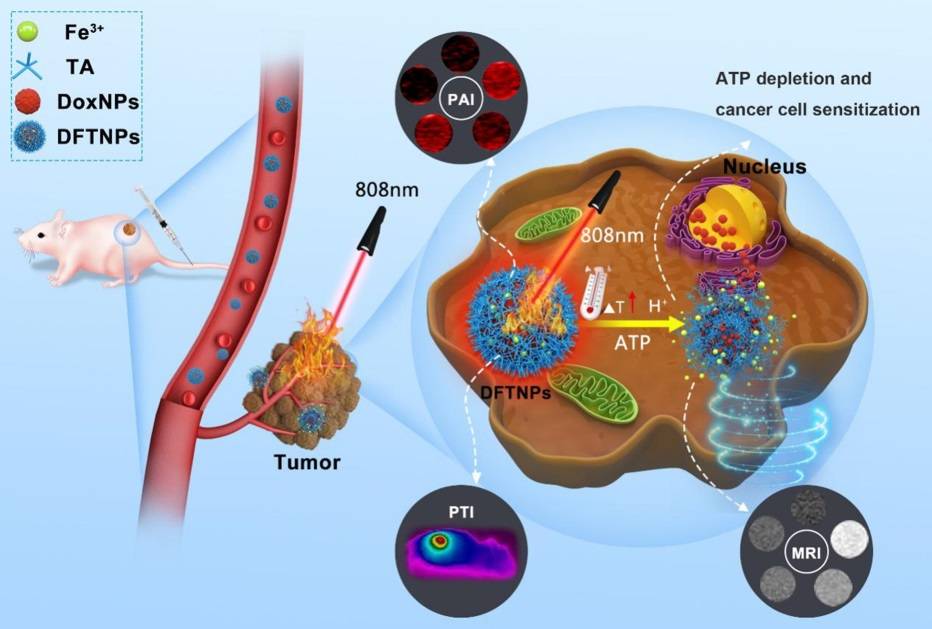
** Schematic illustration of the theranostic performance of as-synthesized DFTNPs as a photothermal platform and in response to ATP/acidic environment in tumor sites.** Upon uptaken by cancer cells, the DFTNPs enabled cellular ATP depletion to increase the chemosensitivity, resulting in simultaneously enhanced cancer cell chemosensitivity, MRI performance and therapeutic efficacy under multimodal imaging guidance.

Herein, we report, for the first time, a rational construction of a minimalist and versatile nanoplatform for enhancing chemo-photothermal synergistic therapy by concurrently achieving pH/ATP-dual responsive drug release and MRI enhancement, ATP-depletion enhanced chemotherapy sensitivity under multiple imaging guidance. As illustrated in **Scheme 1**, the multifunctional theranostic nanoplatform was successfully constructed for tumor growth inhibition with high selectivity, specificity and efficiency, which was based on Fe^3+^/TA modified Dox nanoparticles (designated as DFTNPs). Initially, the DFTNPs could penetrate into the tumor region by the typical enhanced permeability and retention (EPR) effect. DFTNPs showed high stability under physiological conditions, followed by accelerating DOX release in acidic and ATP in the TME. Importantly, the overexpressed ATP degrades the Fe^3+^/TA into Fe^3+^ and TA, accompanied by the depletion of ATP and the increase in chemosensitivity. Especially, the high photothermal conversion efficiency of DFTNPs can elevate the temperature in the tumor region to induce cell death, enabling a chemo-photothermal synergistic therapy with improved chemotherapy sensitivity for combating tumors. Importantly, MRI, PAI, and PTI were integrated into the nanoplatform for guiding and monitoring the therapeutic process. Therefore, the as-prepared minimalist versatile nanoplatform could be promising as on-demand theranostic agents with improved antitumor efficacy for tumor multimodal imaging-guided chemo-photothermal synergistic therapy.

## Methods

### Chemicals and Reagents

Tannic acid, Iron (III) chloride hexahydrate (FeCl_3_·6H_2_O), Dimethyl sulfoxide (DMSO) and trimethylamine (TEA) were purchased from Sigma-Aldrich (St. Louis, MO, USA). Doxorubicin was purchased from Adamas-beta (Shanghai, China). DAPI and penicillin/streptomycin solution were purchased from Beyotime Biotechnology. CCK-8 Assay, Calcein-AM and propidium iodide (PI) were purchased from Dojindo (Japan). All other chemicals used in this work were analytical-reagent grade and used without purification.

### Preparation of DFTNPs

First, DoxNPs were prepared according to the previous report with a minor modification. Briefly, 10 mg Dox·HCl was dissolved in 10 mL DMSO solution. Then 1 mL of TEA was added into the mixture under a moderate stirring at room temperature, converting hydrophilic Dox·HCl into nanosized Dox nanoparticles by removing the hydrochloric acid. Then, 100 μL as-prepared Dox/DMSO solution was poured into 880 μL of high-purity water under violent stirring for 30 min. Next, 10 μL of TA solution (40 mg/mL) and 10 μL of FeCl_3_·6H_2_O (20 mg/mL) were added sequentially to the above solution under continuous ultrasonication(100W, 2 min) followed by neutralization using 1 μM NaOH solution. The obtained product was rinsed with deionized water by centrifugation(10,000 rpm, 10 min) to remove the excess Dox, TA, and FeCl_3_. The nanoparticles were dispersed in distilled water to obtain DFTNPs.

### Characterization of DFTNPs

The morphology of DFTNPs was observed by a transmission electron microscope (JEM-2100F) and a scanning electron microscope (TM4000 PLUS II).

The size distribution and zeta potential of DFTNPs were analyzed by a Laser Particle Size Analyzer System (ZS90, Malvern instrument Ltd, USA).The Fe content in the DFTNPs was determined using Inductively Coupled Plasma Optical Emission Spectrometer (ICP-OES, Optima 8000). The magnetic properties of DFTNPs was determined by Physical Property Measurement System (PPMS-9T, Quantum Design, USA). The UV-Vis-NIR absorption spectra was recorded by a UV-Vis-NIR spectrophotometer (UV-3600, Shimadzu, Japan). A confocal laser scanning microscopy (Nikon A1) was used to obtain live/dead cell staining and intracellular uptake images. The apoptosis status of the cancer cells was evaluated with a flow cytometer (BD LSRFortessa).

### Determination of Dox Encapsulation Efficiency

The standard curve of Dox (dissolved in distilled water) was established using an UV-vis-NIR spectrophotometer at the wavelength of 480 nm. The encapsulation efficiency (EE) of Dox was determined by examining the content of Dox in the supernatant, and EE was calculated according to the formula: EE% = 1- weight of Dox in the supernatant/ total weight of Dox.

### *In Vitro* Dox Release

Dox release performance was determined in a dialysis bag at various pH values (5.5, 6.5, and 7.4) or ATP content (0, 2, and 10 mM) in buffer solution at 37 ℃. Briefly, 5 mL of as-prepared DFTNPs solution in a dialysis bag (MWCO:3500 Da) immersed in 15 mL of a buffer solution with stirring rate at 160 rpm for 120 h. At specific time intervals, 1 mL of 5% glucose solution with different pH values or determining concentrations of ATP was replaced with an equal volume of fresh glucose solution. Then the cumulative release amount of Dox was calculated based on the corresponding absorbance of UV-spectrum at 480 nm and high-performance liquid chromatography (HPLC) according to the standard calibration.

### *In Vitro* Photothermal Effects

DFTNPs were added into 96-well plates and their thermal profiles were measured under irradiation with 808 nm laser at elevated laser irradiation intensities (0.5, 1.0, 1.5, and 2.0 W/cm^2^) with different concentrations (50, 100, 200, 300, and 400 μg/mL) for 10 min. To evaluate the effective components of photothermal characteristics, 200 μL of sample solution of DFTNPs, Fe^3+^/TA solution, DoxNPs, TA, and FeCl_3_ with identical concentration was added into 96-well plates and their thermal profiles were measured under irradiation with 808 nm laser at 2.0 W/cm^2^ for 10 min. The temperature change at different time intervals was recorded by an infrared thermal imaging camera (Fotric 226, Shanghai, China). The distilled water was used as a control at the same conditions. To test the photothermal stability of DFTNPs, the DFTNPs solution was exposed to 808 nm laser and irradiated for repeated heating and cooling cycles.

### Cell Culture

MDA-MB-231 breast cancer cells were used for *in vitro* and *in vivo* evaluations. MDA-MB-231 cells were incubated at 37 ℃ in a humidified atmosphere of 5% CO_2_. The MDA-MB-231 cells were grown in Dulbecco's Modified Eagle's Medium containing 10% fetal bovine serum and 1% penicillin/streptomycin.

### *In Vitro* Cell Viability

A standard CCK-8 cell viability assay was employed to assess the cell toxicity and therapeutic effect of DFTNPs *in vitro*. MDA-MB-231 cells (1×10^4^ cells per well) were plated in 96-well plates overnight. Then, 100 μL of DFTNPs or DoxNPs with various concentrations of Dox (range from 0-65 μg/mL) were added and incubated for another 36 h, 10 μL of CCK-8 solution was added into each well of a 96-well plate to evaluate the cell viability using a microplate reader. To further evaluate the chemo-photothermal synergistic therapeutic effect of DFTNPs, MDA-MB-231 cells were treated with various concentrations of DFTNPs (range from 0-200 μg/mL) for 36 h. Then the cells were exposed to 808 nm laser (2.0 W/cm^2^) irradiation for 10 min and incubated for another 12 h and the cell viability was tested using CCK-8 assay. To evaluate the therapeutic effect of DoxNPs and DoxNPs plus Fe^3+^/TA, MDA-MB-231 cells were cultured with DoxNPs for 4 h, and different concentrations of Fe^3+^/TA were added and incubated for 36 h.

### Cell Apoptosis Assay

The therapeutic efficacy of DFTNPs upon 808 nm laser irradiation was evaluated against MDA-MB-231 cells with the following six groups: control group, control combined with laser irradiation group, DoxNPs only group, DoxNPs combined with laser irradiation group, DFTNPs only group and DFTNPs combined with laser irradiation group. MDA-MB-231 cells were plated in 6-well plates for 12 h to adhere to the plates and then 5% glucose, DoxNPs and DFTNPs (Dox concentration: 50 μg/mL) were added to displace the previous culture medium and subsequently incubate for another 24 h. The plates were treated by the protocols as mentioned above, and the cells were collected after trypsinization. The precipitate was resuspended and stained for 15 min. Finally, cell apoptosis status was detected by flow cytometry. Similarly, the living cells and dead cells were observed by CLSM after costaining with Calcein-AM and PI solution for 15 min.

### Intracellular Endocytosis Analysis

The MDA-MB-231 cells (5×10^4^ cells per dish) were seeded into specific glass culture dishes. The medium was discarded and then replaced by DMEM containing DFTNPs (1 mL, 40 μg/mL). After different incubation intervals (1 h, 2 h, 4 h, and 8 h), the cells were washed thrice and the cell nucleus was stained with DAPI for 15 min. Finally, cellular uptake behavior was observed by CLSM.

### *In Vitro* and *In Vivo* PA Imaging

To test the potential of DFTNPs as a PA imaging contrast agent *in vitro*, different concentrations of DFTNPs were measured using a Vivo LAZR photoacoustic imaging system (VisualSonics, Co., Ltd.). Besides, MDA-MB-231 tumor-bearing Balb/c nude mice were selected to test the PA imaging capability *in vivo*. The mice were injected with DFTNPs (Dox concentration: 5 mg/kg) via the tail vein. The PA signals of the tumor were captured at different time intervals (0, 2, 4, and 24 h). The PA imaging parameters were set as follows: transducer frequency: 21 MHz; PA gain: 46 dB; focal depth: 12 mm.

### *In Vitro* and *In Vivo* MR Imaging

To test the potential of DFTNPs as an MRI contrast agent for applications *in vitro*, different concentrations of DFTNPs were measured using a Siemens MRC40690 3.0 T MR scanner (Erlangen, Germany). MDA-MB-231 tumor-bearing Balb/c nude mice were also used to evaluate the MR imaging capability for applications *in vivo*. The mice were injected with DFTNPs (Dox concentration: 5 mg/kg) via the tail vein. The MRI signals of the tumor were captured at different time intervals (0, 2, 4, and 24 h). The parameters used in the MRI were as follows: fast field echo (repetition time (TR)/echo time (TE)=790/15 ms, slice thickness 3.00 mm, 320 × 320 matrices).

### *In Vitro* and *In Vivo* ATP Analysis

MDA-MB-231 cells were seeded in 12-well plates (2×10^5^ cells per well). Various concentrations of Fe^3+^/TA were added to cells and incubated for 36 h. The cells were washed with 5% glucose and lysed. After counting and normalizing the numbers of cells in each group, the ATP contents were determined by the ATP assay kit according to the manufacturer's instructions. To further determine the intratumoral ATP content, corresponding tumors were digested and lysed, then the ATP content was measured by the ATP assay kit using a microplate reader at the wavelength of 570 nm.

### *In Vivo* Antitumor Effect

MDA-MB-231 xenograft model was established by subcutaneous injection with MDA-MB-231 cancer cells dispersion in PBS (100 μL, 10^7^/mL) at the right backside of each nude mouse. When the tumors increased to 100 mm^3^, the mice were randomly separated into six groups (n=6): control group, laser only group, DoxNPs only group, DFTNPs only group, DoxNPs combined with laser group, DFTNPs combined with laser group. The mice bearing MDA-MB-231 tumor were intravenously injected with 5% glucose, DoxNPs, DFTNPs (Dox concentration: 5 mg/kg). After 24 h, one-half of the tumors were treated by NIR laser irradiation (808 nm, 2 W/cm^2^) for 10 min. The photothermal images and temperatures at different points were recorded by an infrared thermal imaging camera. The body weight and the tumor volume of each mouse were measured every other day. The tumor volume (mm^3^) was calculated by the following formula: V=L×W^2^/2, where the W and L are the minimum and maximum diameter, respectively. The relative volume was calculated as V/V_0_, where V_0_ and V are tumor volume before and after treatment, respectively. The weight of the mice was recorded every other day until 14 d post-treatment.

On the 3rd day, one tumor-bearing mouse in each group was chosen to sacrifice, then the tumors and the major organs including heart, liver, spleen, lungs, kidneys, and brain were collected for hematoxylin and eosin (H&E) staining to examine the histological changes. Proliferating cell nuclear antigen (PCNA) staining was used to evaluate the proliferation condition and terminal deoxynucleotidyl transferase-mediated dUTP-biotin nick and labeling (TUNEL) staining was performed to evaluate apoptosis condition of tumors.

### Toxicity Studies of DFTNPs *In Vivo*

To study the toxicity of DFTNPs *in vivo*, twenty healthy Kunming mice (4-6 weeks) were randomly divided into four groups and injected with DFTNPs (0, 5, 10, and 20 mg/kg). Then the blood samples were collected for the routine blood test and biochemical test before treatment at 30 d. The major organs (including heart, liver, spleen, lungs, kidneys, and brain) were stained with H&E for histological analysis.

### Statistical Analysis

All data were expressed as mean ± standard deviation (SD) and the significance levels among groups' data were evaluated based on one-way ANOVA analysis (*p < 0.05, **p < 0.01, ***p < 0.001).

## Results and Discussion

### Preparation and Characterization of DFTNPs

**Figure 1:**
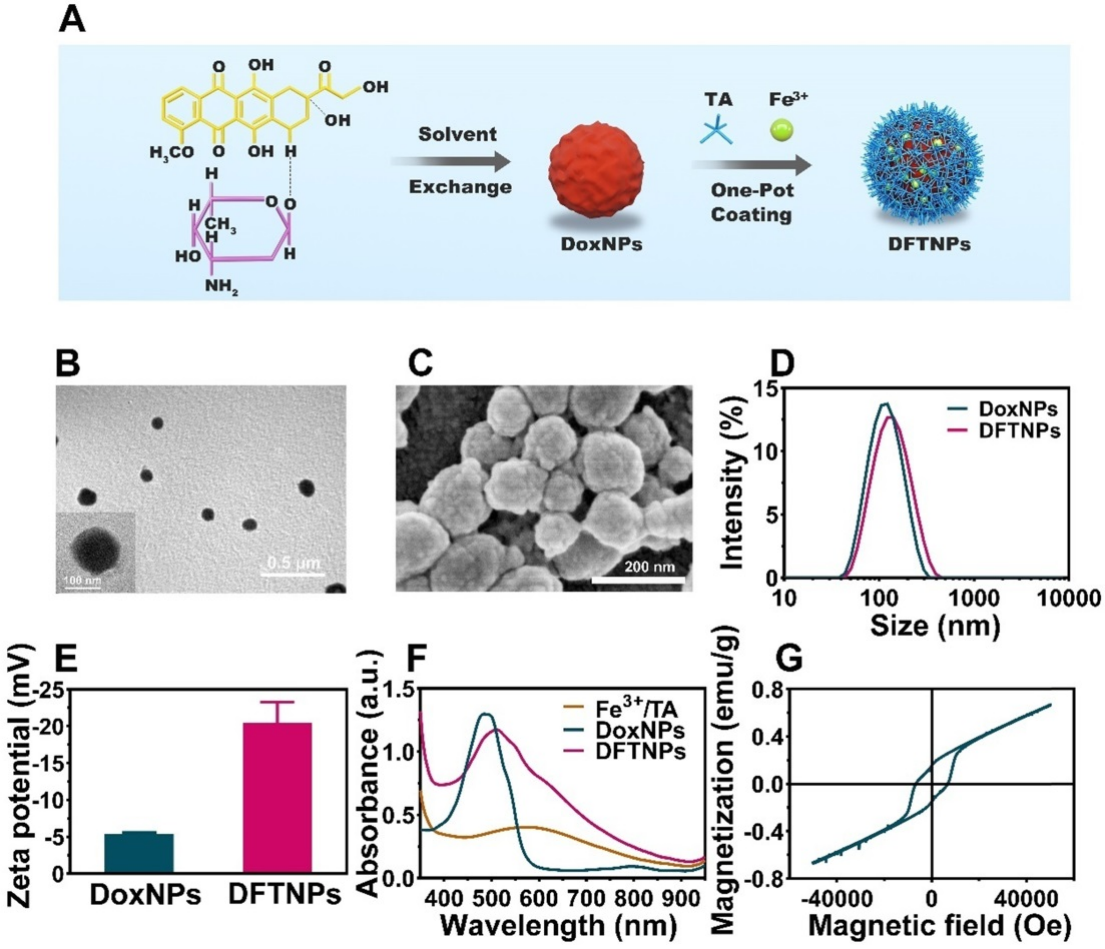
**Composition and structure characterization of DFTNPs. (A)** Schematic illustration of synthetic procedure of DFTNPs. **(B)** TEM image of DFTNPs. **(C)** SEM image of DFTNPs. **(D)** Size distribution of DoxNPs and DFTNPs. **(E)** Zeta ptential of DoxNPs and DFTNPs. **(F)** Absorption spectra of DFTNPs, DoxNPs and Fe^3+^/TA complex. **(G)** Magnetization hysteresis loops of DFTNPs at 500 K range from -60 kOe to +60 kOe.

The synthetic process of DFTNPs was illustrated in **Figure 1**A. TA, a ubiquitous natural polyphenol with diverse chelation ability, can coordinate with different metal irons into a three-dimensional network within seconds in the presence of a substrate 37. As a result, DoxNPs was first prepared as a template via a reprecipitation method 44. Briefly, an aliquot of Dox/DMSO solution was poured into distilled water under intense agitation, allowing for the Dox molecules aggregation and precipitation to form Dox nanocores. Then immediately TA and Fe^3+^ aqueous solution was sequentially added to the above solution to form a Fe^3+^/TA coating network on the surface of Dox nanocores under constant ultrasonic stirring followed by pH neutralization. A lilac color suspension appeared after the addition of TA and Fe^3+^ aqueous solution, while a red color in the suspension of Dox nanocore (**Figure S1**A). The immediate change in appearance prompts the complexation between TA and Fe^3+^ aqueous solution, forming a supramolecular shell enclosing the Dox nano templates. When using nano drugs as particular templates, a drug encapsulation efficiency of 76.3% has been achieved without additional inert drug carriers (such as liposomes 45,46, polymeric micelles 47, polymersomes 48,49, carbon nanotubes 50, mesoporous silica nanoparticles 33,51, as well as no need of removing the templates. The particular drug as templates also contributes to the minimalist design of this theranostic nanosystem. Transmission electron microscope (TEM) image, scanning electron microscope (SEM) and elemental mapping results showed that the spherical DFTNPs with an average diameter of proximately 120 nm (**Figure 1B-C,** and** S2**), which is consistent with the dynamic light scattering (DLS) measurements (**Figure 1D**). The DLS results also revealed that DFTNPs had a hydrodynamic diameter of 121.17 ± 1.07 nm, which was 13 nm larger than that of DoxNPs (108.53 ± 5.62 nm), as a result of the Fe^3+^/TA assembly coating onto the DoxNPs nanocore (**Figure 1D** and **Figure S3**). The successful coating of Fe^3+^/TA assembly was also reflected by the Zeta potential which changes from -5.5 ± 3.7 mV to -21.5 ± 5.3 mV (**Figure 1E**). The polydispersity index (PDI) of DFTNPs was around 0.148, indicating a narrow size distribution. Moreover, the particle size was examined in 120 h observations and no obvious changes were observed (**Figure S4**). To further validate the coating results, UV/Vis absorption spectra were carried out to detect the Fe^3+^/TA complex. In comparison to individual components, new absorbance peaks at around 560-600 nm in both Fe^3+^/TA complex and DFTNPs were observed (**Figure 1F**), indicating the success coordination between TA and the Fe^3+^ 52. The UV/Vis absorption spectra also showed that DFTNPs retained the characteristic absorption peak of Dox at 480 nm, while the Fe^3+^/TA complex exhibited no such a characteristic absorption peak, suggesting the successful DFTNPs synthesis templated DoxNPs. The Dox encapsulation efficiency was calculated to be around 76.3% according to the standard curve of Dox under UV/Vis absorption spectra, respectively (**Figure S5-6**). Moreover, the high-performance liquid chromatography (HPLC) showed a similar Dox encapsulation efficiency compared with the UV/Vis results (**Figure S7**). The DFTNPs dispersed well in physiological conditions with an evident Tyndall phenomenon in buffer solution (**Figure S1B**). Inductively coupled plasma (ICP) showed that the Fe encapsulation efficiency was 43.2%. The examination of magnetic properties by Physical Property Measurement System (PPMS) confirmed the paramagnetic behavior of DFTNPs, indicating the success loading of Fe^3+^ and the potency of DFTNPs as a contrast agent for MRI (**Figure 1G**).

### *In Vitro* Drug Release and MRI profile

**Figure 2:**
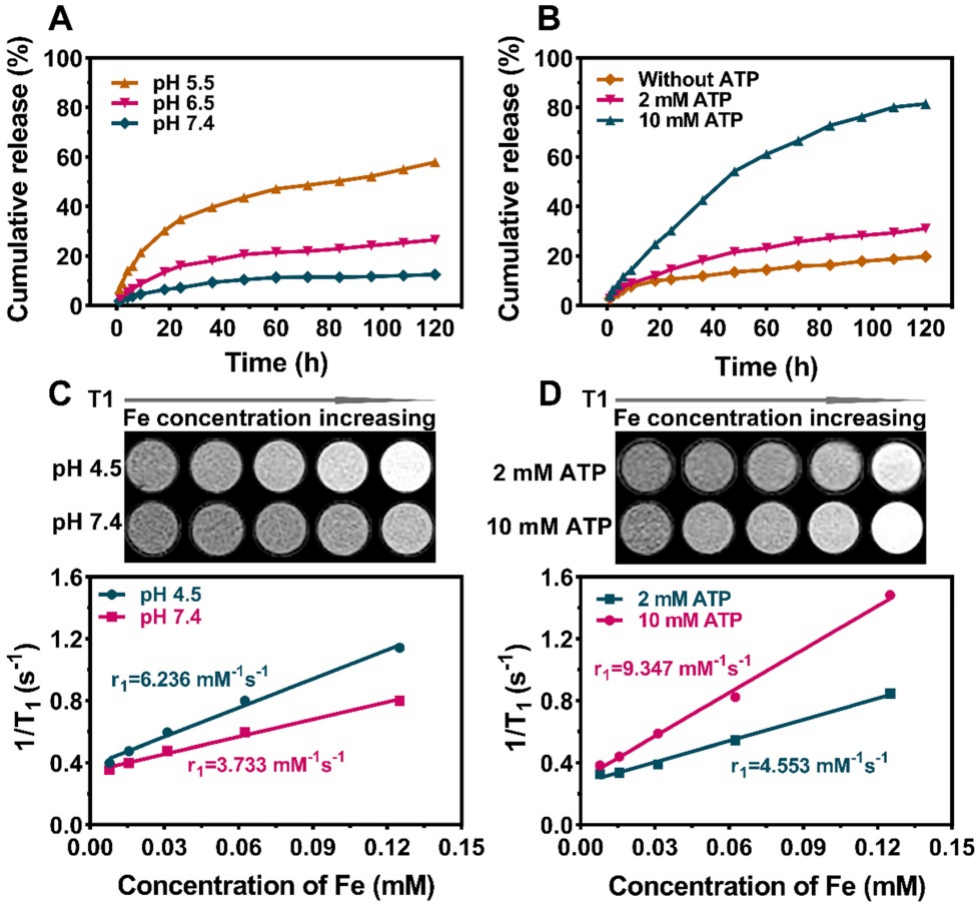
***In vitro* Dox Release and T_1_-weighted MRI imaging performance under different concentrations of ATP and different values of pH. (A)** Cumulative Dox release from DFTNPs under different pH values (7.4, 6.5, 5.5). **(B)** Cumulative Dox release from DFTNPs under different concentrations of ATP (10 mM, 2 mM and 0 mM). **(C)** *In vitro* T_1_-weighted images and corresponding T_1_ relaxation rate (r_1_) of DFTNPs under different pH values (pH=4.5, 7.4). **(D)** *In vitro* T_1_-weighted images and corresponding T_1_ relaxation rate (r_1_) of DFTNPs under different concentrations of ATP (2 mM, 10 mM).

The stability of DFTNPs is an important but arduous challenge based on the coordination of Fe^3+^/TA drug delivery systems due to the vulnerability of non-covalent interactions. Thus, the stability of DFTNPs was investigated in different buffer solutions (pH 7.4, 6.5, and 5.5) to imitate ambient physiological and tumor environment, respectively. The results of drug release *in vitro* showed that no more than 15% leakage of Dox was found during neutral incubation (pH 7.4), which is consistent with the fact that DFTNPs are stable in physiological conditions. In contrast, a burst release of Dox took place in an acidic environment, amounting to 58% at pH 5.5 and 26% at pH 6.5 across the 120 h period, respectively (**Figure 2A**). These results suggested a certain acid-responsive Dox release properties of DFTNPs. Moreover, ATP could in principle competitively coordinate with Fe^3+^ and cause the crush of DFTNPs, facilitating the release of Dox subsequently. Therefore, the potential effect of ATP on the Dox release was also investigated. As an aspect, the Dox release from DFTNPs was faster in the presence of ATP than DFTNPs without ATP, confirming that ATP facilitated to drive Dox release (**Figure 2B**). All the results demonstrated that DFTNPs was a desirable system for “smart” accelerating Dox release in tumor sites with a minimal release in the circulation. It has been reported that nanoparticles based on the Fe^3+^ and TA interaction had substantial T_1_-weighted MR imaging capability. Encouraged by the pH/ATP dual-responsive drug release behavior of the DFTNPs, their MRI properties were investigated in the presence of ATP (2 mM and 10 mM), acidic environment (pH 4.5) and physiological environment (pH 7.4) at room temperature. DFTNPs exhibited a relatively small relaxation rate (r_1_ value) in the neutral condition (pH 7.4) without ATP, which relaxation rate was measured to be 3.733 mM^-1^ s^-1^ and dramatically increase to 4.553 mM^-1^ s^-1^ and 9.347 mM^-1^ s^-1^ under the ATP concentration of 2 mM and 10 mM, respectively (**Figure 2C-D**). A substantial increase in the r_1_ value of 6.236 mM^-1^ s^-1^ was observed after soaking the DFTNPs in acid solutions (pH 4.5) without ATP addition. Moreover, the signal intensity of T_1_-weighted MRI was significantly enhanced when the concentration of Fe and ATP increased, along with the decrease in pH. Compared to the current existing NDDS, the DFTNPs holds unparalleled advantages that maintain high stability under physiological conditions, followed by accelerating drug release and enhancing MRI properties in acidic and ATP-rich TME.

### *In Vitro* Photothermal Effect and PAI profile

**Figure 3:**
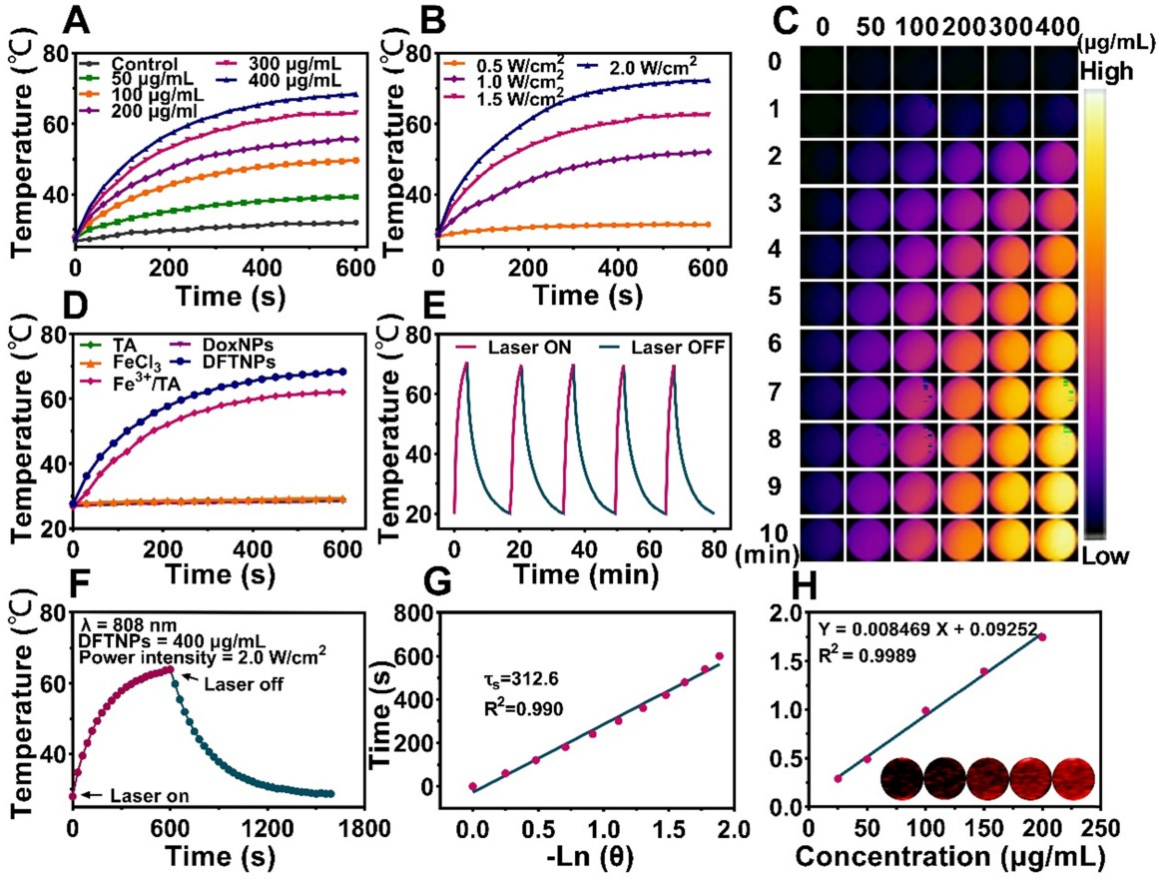
***In vitro* photothermal and photoacoustic imaging effect of DFTNPs. (A)** Photothermal curves of DFTNPs with 808 nm laser irradiation (2.0 W/cm^2^, 10 min) at different concentrations (50, 100, 200, 300 and 400 μg/mL). **(B)** Photothermal curves of DFTNPs dispersed in aqueous solution irradiated under increased power densities (0.5, 1.0, 1.5 and 2.0 W/cm^2^). **(C)** Infrared thermal images *in vitro*. **(D)** Photothermal curves of the aqueous dispersion of DFTNPs, DoxNPs, Fe^3+^/TA complex, TA and ferric chloride. **(E)** Photothermal performance of the aqueous dispersion of DFTNPs under 808 nm laser irradiation at the power intensity of 2.0 W/cm^2^ for periods and the laser was cut off when the temperature tended to be stable. **(F)** The temperature changes of DFTNPs solution (400 µg/mL) under laser irradiation (808 nm, 2 W/cm^2^) for 600 s followed by the cooling period. **(G)** The time constant for heat transfer calculated from the cooling period. **(H)** *In vitro* PA imaging intensities of DFTNPs with various concentrations.

A characteristic feature of DFTNPs was their thermal effect under the NIR irradiation, which could be applied to achieve chemo-photothermal synergistic therapy. The DFTNPs dispersion was continuously irradiated with 808 nm laser at the various concentrations for 10 min. The photothermal curves exhibited a concentration-dependent and irradiation time-dependent manner (**Figure 3A, S8**). Following 10 min exposure with increased laser power from 0.5 to 2.0 W/cm^2^, the values of temperature rose from 31.4 ℃ to 72.3 ℃, respectively. Moreover, the temperature elevation could be finely enhanced at increased power intensity (**Figure 3B**). Meanwhile, PTI performance of various DFTNPs concentrations was also presented (**Figure 3C**). These results provided strong evidence that DFTNPs could act as an efficient photothermal agent for the ablation of the tumor. The photothermal character of DFTNPs encouraged us to further explore the heating properties of various components, including DoxNPs, DFTNPs, TA solution, ferric chloride solution and Fe^3+^/TA complex solution at the same concentration. Only DFTNPs and Fe^3+^/TA complex showed a significant increase in temperature, opposite to the negligible variation for the other three samples (**Figure 3D**). Based on these results, we can infer that the photothermal effect of DFTNPs is strongly correlated with the coordination between TA and Fe^3+^. Furthermore, there was no distinct attenuation on the photothermal effect of DFTNPs during five laser on-off irradiation cycles, indicating high photothermal stability as a photothermal agent for efficient tumor treatment (**Figure 3E**). The photothermal conversion efficiency (*η*) of DFTNPs was determined to be ~38.7% based on the maximum temperature change (∆T_max_) and the time constant for heating transfer (*τs*) (**Figure 3F-G**), which was much higher than Au (21%), Cu_2-x_Se (22%) and Cu_9_S_5_ (26%) according to previous reports, indicating the high photothermal conversion efficacy of DFTNPs for PTT 53-55.

PA imaging is a booming imaging modality with high depth penetration and spatial/temporal resolution for guiding-drug delivery, surgery and diagnosis 31,56,57. Based on this favorable photothermal conversion efficiency in the NIR region, we can infer that DFTNPs have excellent photoacoustic imaging effects. Thus, the dispersion of DFTNPs was subjected to the full wavelength scanning from 680 nm to 970 nm laser under the PA imaging system. It was found that the wavelength at 700-760 nm was the optical bio window for contrast-enhanced PA imaging (**Figure S9**). On account of the PA images obtained at longer wavelengths had higher tissue penetrating capability, the PA imaging was excited and assessed at 760 nm. Moreover, the PA signal strengthened proportionally to the increased DFTNPs concentrations ranging from 25 μg/mL to 200 μg/mL at 760 nm laser excitation (r^2^ = 0.9989) (**Figure 3H**), suggesting that DFTNPs as an efficient photoacoustic imaging agent hold a great promise for biomedical applications.

### Cytotoxicity Assay

**Figure 4:**
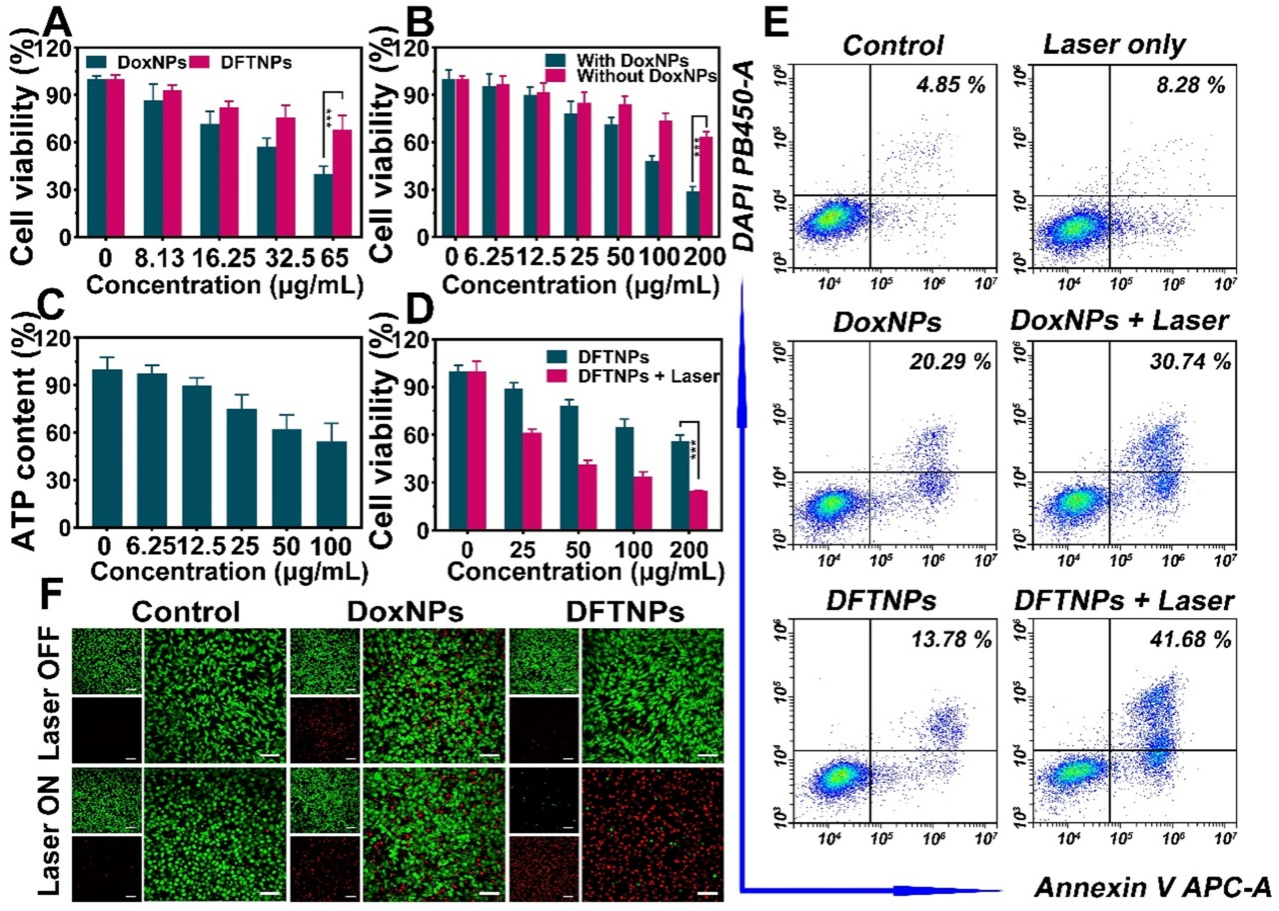
***In vitro* synergistic therapy against MDA-MB-231 cells. (A)** Cell viability of MDA-MB-231 cells after treated with DoxNPs or DFTNPs at various concentrations after 36 h incubation. **(B)** Cell viability of MDA-MB-231 cells treated with or without DoxNPs at various concentrations of Fe^3+^/TA. **(C)** Intracellular ATP content treated with various concentrations of Fe^3+^/TA after standardization with cell number. **(D)** Cell viabilities after exposure to 808 nm laser irradiation under different concentrations. **(E)** Flow cytometry analysis of apoptosis after various treatments. **(F)** CLSM images of Calcein-AM and PI costained MDA-MB-231 cells after coincubation with DFTNPs for 4 h followed by various treatments. The scale bars are 50 μm.

The chemotherapeutic effect of DoxNPs and DFTNPs was then evaluated. After co-incubation with MDA-MB-231 cells for 36 h, the cytotoxicity of both DoxNPs and DFTNPs appeared in a dose-dependent manner (**Figure 4A**). DFTNPs also showed weaker therapeutic efficacy than that of DoxNPs at the same concentration of Dox due to the incomplete drug release in DFTNPs. Interestingly, DoxNPs had higher chemotherapeutic efficacy with the adding of Fe^3+^/TA compared to that of free DoxNPs, and the cell viability decreased with the increasing concentration of Fe^3+^/ TA (**Figure 4B**). Meanwhile, the ATP content was depleted by treating MDA-MB-231 cells with Fe^3+^/TA after standardization of the numbers of cancer cells (**Figure 4C**). Considering the ATP-depletion can enhance the cell chemosensitivity and thus improve the therapeutic effect of chemotherapeutic drugs, these studies demonstrated that the DFTNPs hold great promise for enhancing the therapeutic effect of Dox by depleting the intracellular ATP.

In virtue of the photothermal effect, DFTNPs were also served as photothermal agents for treating tumors. Thus, breast cancer, as a representative superficial tumor in the clinic, was chosen as the treatment model, which is sensitive to Dox in various preclinical studies. After co-incubation with MDA-MB-231 cells for 36 h, the cell viability still maintained around 60% at the concentration of DFTNPs reaching 200 μg/mL (**Figure 4D**). Upon exposure to 808 nm laser, the cell viability decreased sharply, revealing the photothermal toxicity of DFTNPs. Especially, such a PTT-induced therapeutic effect of DFTNPs was dose-dependent after 808 nm laser irradiation at 2.0 W/cm^2^ for 10 min. Furthermore, flow cytometry was carried out to test the apoptosis rate of treated cells (**Figure 4E**) and corresponding quantitative analysis was presented (**Figure S10**). The laser only group showed no obvious cytotoxicity compared with the control group. Besides, the DFTNPs showed weaker therapeutic effect compared to DoxNPs, which is consistent with the CCK-8 results. Furthermore, the DFTNPs combined with 808 nm laser irradiation exhibited the highest cytotoxicity compared to other treatments, demonstrating enhanced therapeutic efficacy of chemotherapy and photothermal synergistic therapy. The therapeutic effect of DFTNPs against MDA-MB-231 cells was also evaluated by CLSM (**Figure 4F**). DFTNPs induced the maximum cell death under 808 nm laser irradiation, which was consistent with the results of both flow cytometry and CCK-8 assay.

### Cellular Uptake

Efficient transport and release of Dox into tumor cells are considerable factors affecting the therapeutic efficacy of DFTNPs. CLSM examination of MDA-MB-231 cells incubated with DFTNPs in parallel for different times (1 h, 2 h, 4 h, and 8 h) showed a uniform red fluorescence of Dox in tumor cells (**Figure S11-12**). The fluorescence signal in MDA-MB-231 cells increased dramatically with the prolongation of incubation time and more Dox was released into the nucleus, indicating that the endocytosis process of DFTNPs was time-dependent.

### *In Vivo* PAI/MRI/PTI Profile

**Figure 5:**
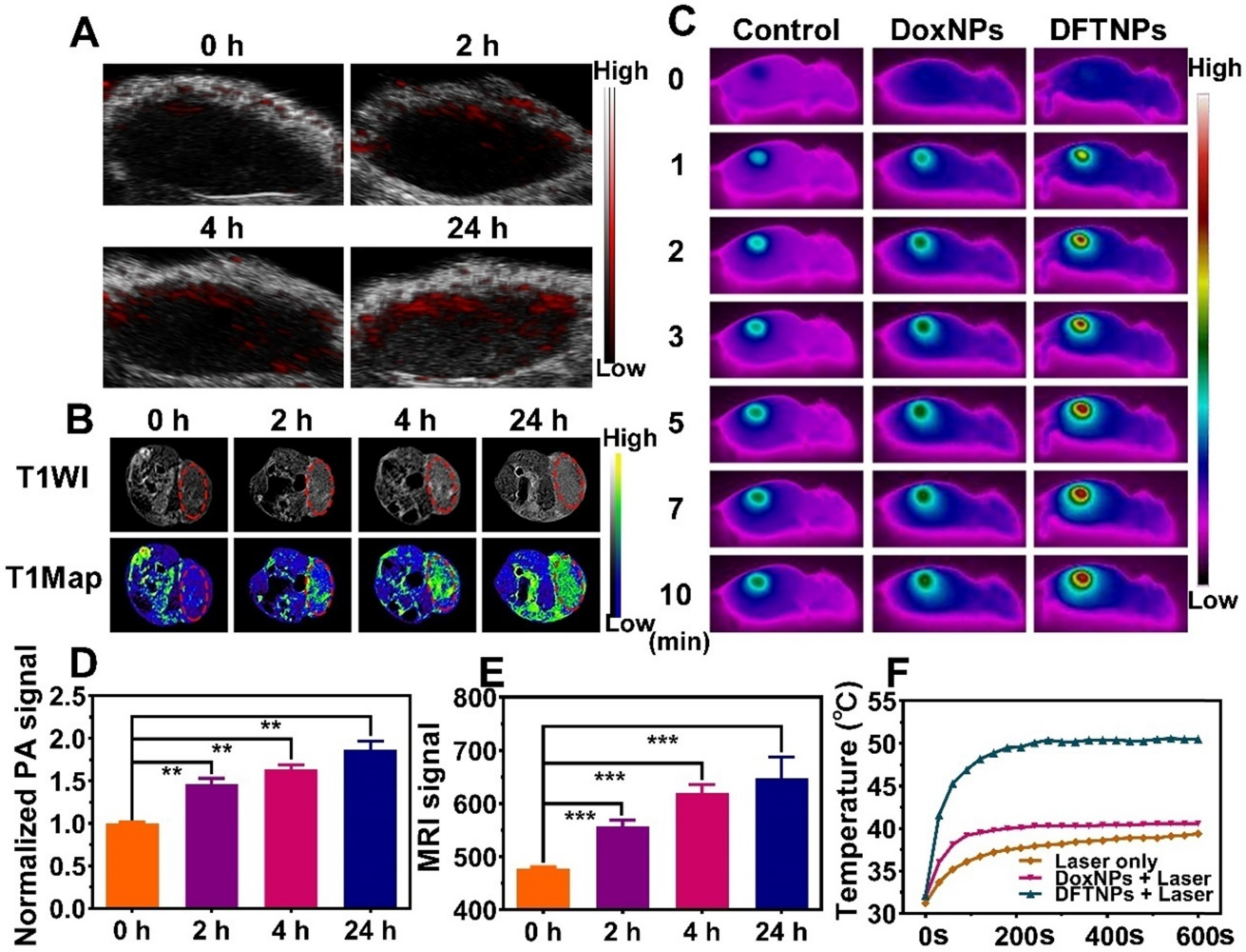
***In vivo* multiple imaging and corresponding quantification analysis. (A)** *In vivo* PA images of MDA-MB-231 tumor-bearing mice after intravenous injection of DFTNPs. **(B)** *In vivo* T_1_-weighted images and T_1_-mapping images of MDA-MB-231 tumor-bearing mice after intravenous injection of DFTNPs. **(C)** Photothermal images of tumor-bearing mice post various treatments. **(D-E)** Quantification analysis of normalized PA imaging signal and T_1_-weighted images within the tumor region at corresponding time points. **(F)** Temperature increase behaviors of tumor tissues in the mice post various treatments.

Considering the strong photothermal effect and the involvement of Fe^3+^ ions as a T_1_-weighted MRI contrast, the potential of the DFTNPs as PAI/MRI/PTI contrast for tumor imaging was evaluated in MDA-MB-231 tumor-bearing mice. Mice bearing MDA-MB-231 tumors were injected with DFTNPs via the tail vein then imaged by a PA imaging system at predetermined times. The results showed that the DFTNPs accumulated at the tumor sites in a time-dependent manner, and the intratumoral PA imaging signals were significantly strengthened (**Figure 5A**). Correspondingly, the quantitative intensity analysis also revealed that the PA imaging signal reached its peak at 24 h post-injection (**Figure 5D**). The enhancement in PA signal among the tumor-bearing mice manifested the desirable performance DFTNPs as a PA imaging contrast agent *in vivo*. Then T_1_-weighted MRI performance of DFTNPs was further evaluated in MDA-MB-231 tumor-bearing mice. As shown in the MR images, the signal intensity of tumor sites was relatively low before the injection of DFTNPs. An evident intensification of T_1_-weighted MR images in the tumor region gradually increased over time. Consistent with the PAI results, an obvious MRI enhancement in the tumor region was achieved at 24 h post-injection of DFTNPs (**Figure 5B**). Compared with pre-injection tumor tissue, the quantitative MRI signal intensities also showed an obvious time-dependent brightening effect in 24 h observations (**Figure 5E**). A substantial enhancement of quantitative MRI signal intensity in comparison with non-tumor tissue was also observed in the T_1_-weighted MR images (**Figure S13**). The high accumulation of DFTNPs into tumors could be mainly attributed to the EPR effect, following by the release of Fe^3+^ in the TME, which maximizes the interaction between Fe^3+^ and water molecules to strengthen the MRI signal intensity.

To further investigate the PTI performance *in vivo*, the mice were intravenously injected with DFTNPs, DoxNPs, 5% glucose and applied to 808 nm laser irradiation (2.0 W/cm^2^, 10 min) at 24 h post-injection. Meanwhile, the temperature change in the tumor region was recorded with an infrared thermal camera (**Figure 5C and 5F**). The tumor temperature increased by 8.0 °C in the control plus laser group and 9.2 °C in DoxNPs combined with an 808 nm laser group, whereas the temperature of tumor site in DFTNPs combined with 808 nm laser group increased by 19.0 °C during the irradiation period (**Figure S14**), which was able to induce hyperthermia and eradicate the tumors. These findings demonstrated that the as-prepared DFTNPs possessed excellent multimodal imaging and efficient tumor accumulation ability for the applications *in vivo*.

### *In Vivo* Anticancer Activity

**Figure 6:**
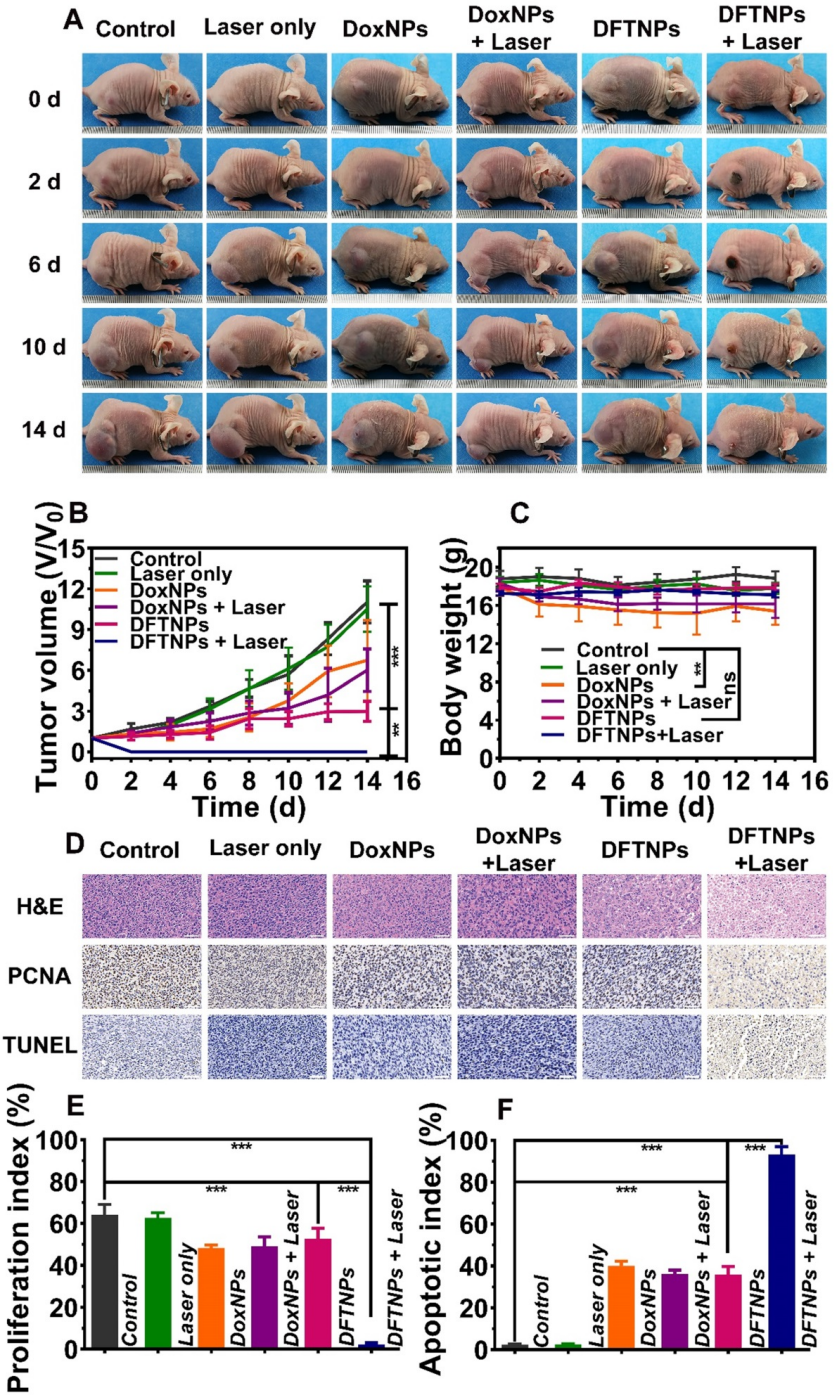
***In vivo* chemo-photothermal synergistic therapy. (A)** Photographs of MDA-MB-231 tumor-bearing mice of six groups during 14 d period treatments. **(B)** Relative tumor volume after various treatments of six groups. **(C)** Body weight change in various groups. **(D)** H&E, PCNA and TUNEL staining on tumor sections collected from MDA-MB-231 tumor-bearing mice after various treatments. The scale bar is 50 μm. **(E)** Quantitative proliferation index of tumors from different groups. **(F)** Quantitative apoptotic index of tumors from different groups.

The excellent PTT and chemotherapy efficiency *in vitro* encouraged us to pursue their anti-tumor application *in vivo*. The mice were intravenously injected with DFTNPs, DoxNPs and 5% glucose, then one-half of the tumors were applied to 808 nm laser irradiation (2.0 W/cm^2^, 10 min) at 24 h post-injection. In terms of the tumor volume index, the single employment of 808 nm laser irradiation (2 W/cm^2^, 10 min) exhibited a negligible therapeutic effect on tumor therapy (**Figure 6A-B**). Comparatively, the mice treated with DoxNPs or DFTNPs only could partially slow down the tumor growth, with the tumor inhibition rate of 45% and 81%, respectively (**Figure S15**). DFTNPs showed superior anti-tumor effect than DoxNPs, which can be ascribed to the depletion of ATP for the enhancement of chemosensitivity (**Figure S16**). Of note, the best tumor-growth inhibition efficiency was achieved in the group receiving DFTNPs combined with 808 nm laser irradiation, where the tumors were thoroughly eliminated without relapse during the treatment period, confirming that the combination of chemotherapy and photothermal therapy could completely eradiate the tumors in our observation period. All the results showed that DFTNPs could passively target to tumor tissues. Acidic pH and ATP in tumor microenvironment triggered the accelerating release of Dox in the tumor region, as well as the local heating effect of Fe^3+^/TA, which contributed to an ideal anti-tumor activity. Moreover, no significant loss in body weight was recorded during the treatment period in the DFTNPs group and DFTNPs plus laser irradiation group (**Figure 6C**), while the DoxNPs and DoxNPs plus laser irradiation group showed significant weight loss. The body weight changes suggested the severe side effects induced by DoxNPs and improved biosafety after coating Fe^3+^/TA complex on DoxNPs.

**Figure 7:**
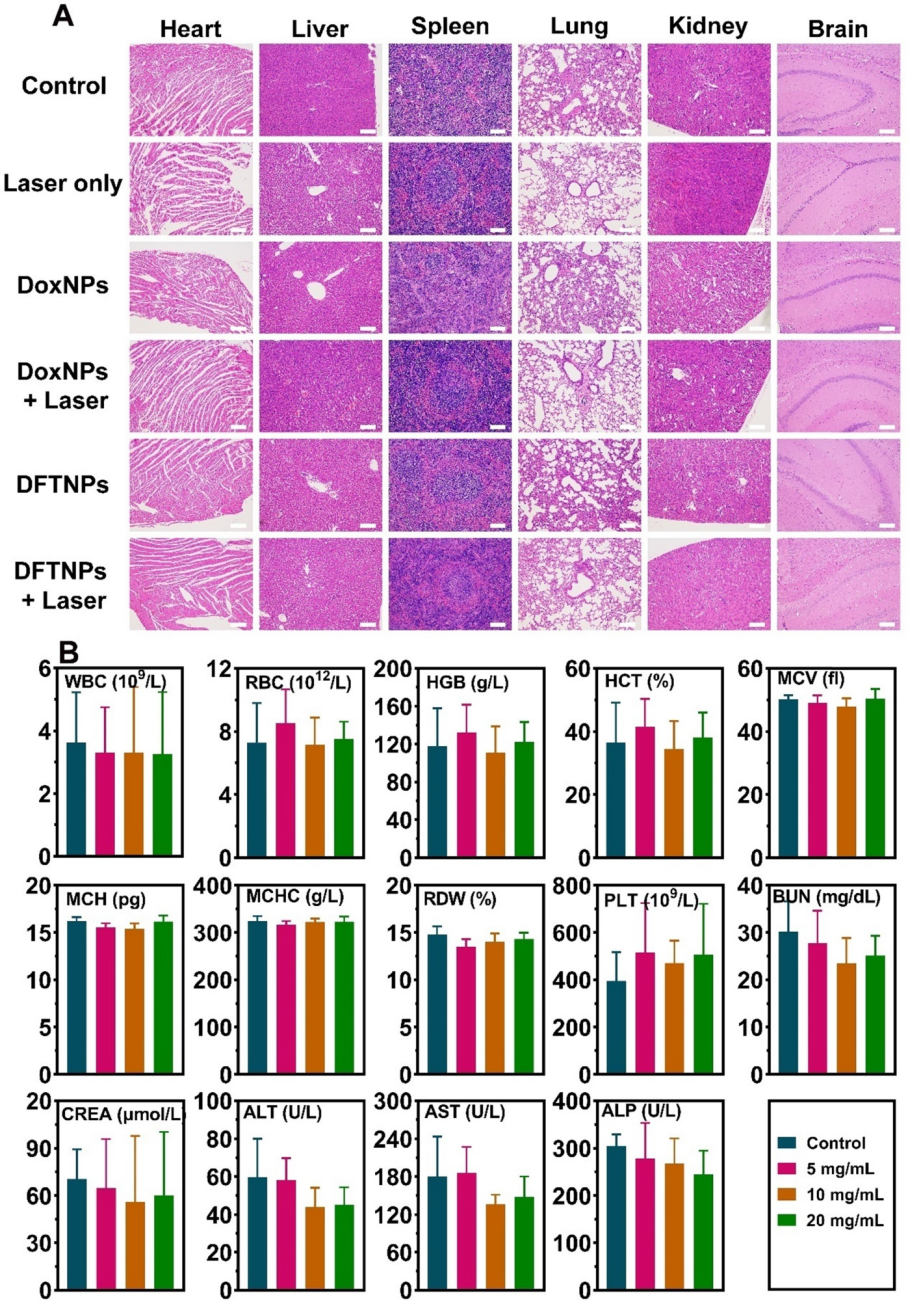
***In vivo* histocompatibility of DFTNPs. (A)** Histological sections of the heart, liver, spleen, lungs, kidneys, and brain from the mice after various treatments. Scale bars: 100 μm. **(B)** Histograms depicting variations in the routine blood test and blood biochemistry treated by DFTNPs with different doses over 30 d.

On the 3^rd^ day, one mouse in each group was sacrificed to excise the tumor and major organs. H&E staining of tumors showed that severe necrosis was observed in DFTNPs plus laser irradiation group, while only moderate damage appeared in DFTNPs, DoxNPs, and DoxNPs plus laser irradiation groups (**Figure 6D**). In contrast, little damage in tumors was observed in control or control plus laser group, indicating that the simple laser irradiation could not induce evident damage to cancer cells. Moreover, the proliferation and apoptosis rates of tumors were further quantified by PCNA and TUNEL assay. Quantitative analysis of PCNA showed the proliferating rate of 2.3% in DFTNPs plus laser irradiation group, much lower than DoxNPs plus laser group (48.4%) and DFTNPs only group (52.7%) (**Figure 6E**). Similarly, TUNEL results showed the maximum inhibition rate of 93.2% in DFTNPs plus laser group, more effective than DoxNPs plus laser group (36.3%) and DFTNPs only group (35.9%) (**Figure 6F**). In contrast, the control group showed a negligible apoptosis rate of approximately 2.4%. The above results demonstrated the efficient drug delivery properties into the tumor region, as well as their chemotherapeutic effect combined with photothermal therapy. Thus, DFTNPs can serve as a versatile theranostic nano platform for multimodal imaging-guided chemo-photothermal synergistic therapy. Moreover, H&E staining was carried out to stain the section of the heart, liver, spleen, lungs, kidneys, and brains of various groups (**Figure 7A**). The tissue sections showed no obvious pathological damage or inflammatory lesion during the treatments, which further indicated the biosafety of DFTNPs.

To further study the biological safety of DFTNPs *in vivo*, various biochemical indexes and pathological results were examined on healthy Kunming mice treated with DFTNPs at 30 d post-injection and the mice without treatment as control. H&E staining was used to stain the heart, liver, spleen, lungs, kidneys, and brain among different concentrations of DFTNPs treated mice. No obvious pathological damage or inflammatory lesion was observed (**Figure S17**), further indicating undetectable toxicity to mice at our tested dose regardless of the short-time and long-time study. The values of routine blood count (RBC, WBC, PLT, etc) and blood biochemistry (ALT, AST, ALP, ALB, etc) all showed a negligible difference from the values in healthy Kunming mice among different therapeutic doses (**Figure 7B**).

## Conclusions

In summary, this work reported a rational construction of a minimalist and versatile nano platform for enhancing chemo-photothermal synergistic therapy by concurrently achieving pH/ATP-dual responsive drug release and MRI enhancement, ATP-depletion enhanced chemotherapy sensitivity under multiple imaging guidance. After surface Fe^3+^/TA engineering, DFTNPs was featured with high biocompatibility. Because of their highly ATP-depletion of Fe^3+^/TA and abundant ATP in tumor cells, DFTNPs were systematically administrated to deplete endogenous ATP for improving the chemosensitivity of Dox. Importantly, based on the high photothermal performance and pH/ATP responsive drug release properties of DFTNPs, both TME-responsive chemotherapy and photothermal therapy were independently introduced to achieve chemo-photothermal synergistic therapy without reoccurrence in our observation period. Specifically, DFTNPs with pH/ATP responsive MRI enhancement improved the signal-to-noise ratio, making the targeted tumors easily to be distinguished from normal tissue. Last but not least, as excellent MRI, PAI, and PTI multimodal contrast agents, DFTNPs acted as multifunctional theranostic agents can achieve multimodal imaging guidance for synergistic chemosensitization/chemotherapy/photothermal therapy strategy with minimal components.

## Supplementary Material

Supplementary figures.

## Abbreviations

NDDS: nonosized drug delivery systems
TME: tumor microenvironment
ATP: adenosine triphosphate
PTT: photothermal therapy
MRI: magnetic resonance imaging
PAI: photoacoustic imaging
PTI: photothermal imaging
EPR: enhanced permeability and retention
DFTNPs: Fe^3+^/TA modified Dox nanoparticles
DMSO: dimethyl sulfoxide
TEA: trimethylamine
PI: propidium iodide
HPLC: high performance liquid chromatography
CCK-8: cell counting kit-8
DMEM: dulbecco's modified eagle medium
CLSM: confocal laser scanning microscope
H&E: hematoxylin and eosin
PCNA: Proliferating cell nuclear antigen
TUNEL: terminal deoxynucleotidyl transferase-mediated dUTP-biotin nick and labeling
SD: standard deviation
TEM: transmission electron microscope
SEM: scanning electron microscope
DLS: dynamic light scattering
PDI: polydispersity index
ICP: inductively coupled plasma
PPMS: physical property measurement system
