# Supplementary material for: TME-activatable theranostic nanoplatform with ATP burning capability for tumor sensitization and synergistic therapy
Source: Theranostics. 2020 May 25;10(15):6987–7001. doi: 10.7150/thno.44569 (PMC7295044; doi:10.7150/thno.44569)
Supplement: Supplementary file 1 — Supplementary figures. [file thnov10p6987s1.pdf]

Supporting information for Theranostics

TME-activatable theranostic nanoplatform with ATP burning capability for tumor sensitization and synergistic therapy

Yuanli Luo[1#], Bin Qiao[1#], Ping Zhang[1], Chao Yang[2], Jin Cao[1], Xun Yuan[3], Haitao Ran[1], Zhigang Wang[1], Lan Hao[1], Yang Cao[1], Jianli Ren[1*], Zhiyi Zhou[4*]

1. Ultrasound Department of the Second Affiliated Hospital of Chongqing Medical University, Chongqing Key Laboratory of Ultrasound Molecular Imaging, Chongqing, 400010, P. R. China.

2. Radiology Department of Chongqing General Hospital, University of Chinese Academy of Sciences, Chongqing 400014, P. R. China.

3. Ophthalmology Department of the Second Affiliated Hospital of Chongqing Medical University, Chongqing, 400010, P. R. China.

4. General Practice Department of Chongqing General Hospital, University of Chinese Academy of Sciences, Chongqing 400014, P. R. China.

[#]Yuanli Luo and Bin Qiao are co-first authors who contributed equally to this work.

Corresponding author: renjianli@cqmu.edu.cn (Jianli Ren), zzy16966@163.com (Zhiyi Zhou)

Part A: In Vitro Calculation of Photothermal Conversion Efficiency

The photothermal conversion efficiency (η) of DFTNPs was calculated following the method reported by previous report[1,2]. Briefly, 200 μL of DFTNPs dispersion at a concentration of 400 μg/mL in a 96-well plate were irradiated by 808 nm laser at 2 W/cm^2, and then the laser was turned off when the temperature of the solutions increased to a steady-state. The whole process of dispersion's temperature change was recorded by an infrared thermal imaging camera (Fotric 226, Shanghai, China). The η was calculated based on the following formula：

(1) $\eta = \frac{\mathrm{hA}\,\Delta \mathrm{T}_{\mathrm{Max}} - \mathrm{Qs}}{\mathrm{I}\,(1-10^{-\mathrm{A}_{\lambda}})}$

(2) $\tau_s = \frac{\sum_{\mathrm{i}} \mathrm{m}_{\mathrm{i}} \mathrm{C}_{\mathrm{p,i}}}{\mathrm{hA}}$

From formula (1), η was regarded as conversion efficiency value, ΔT_{max} was regarded as the temperature change between the highest and lowest equilibrium temperature. Qs was related to the light absorbance of distilled water here, which was regarded as the heat energy produced by the distilled water. I was regarded as the laser power density. A_{λ} was regarded as the absorbance of the dispersion at 808 nm according to the UV-Vis spectrum. The value of hA could be calculated from the formula (2).

From equation (2), the mass of water (m) and the heat capacity of water (C) were regarded as the mass of solution and its heat capacity, respectively. The time constant (τ_s) of the heating system was determined by applying the linear time-dependent data collected during the cooling period.

Herein, m was 200 mg, C was 4.2 J/(g·K) and τ_s of DFTNPs was determined to be 312.6 s as shown in **Figure 3f.** According to the equation (2), the hA was calculated to be 2.69×10^{-3} W/K.

η value of DFTNPs was subsequently calculated in detail. ΔT_{max} value was 36℃ as shown in **Figure 3g.** Qs here was measured to be 1.68 mW. I was the laser power density that is 2 W/cm^2. A_λ was the absorbance of DFTNPs at 808 nm in the UV-Vis spectrum which was determined to be 0.057. According to equation (1), the η of DFTNPs was calculated to be 38.7%.

References

1. Liu T, Zhang M, Liu W, Zeng X, Song X, Yang X, et al. Metal ion/tannic acid assembly as a versatile photothermal platform in engineering multimodal nanotheranostics for advanced applications. ACS Nano. 2018; 12: 3917-3927.
2. Liu Y, Ai K, Liu J, Deng M, He Y, Lu L. Dopamine-melanin colloidal nanospheres: an efficient near-infrared photothermal therapeutic agent for in vivo cancer therapy. Adv Mater. 2013; 25: 1353-1359.

Part B: Supporting Information

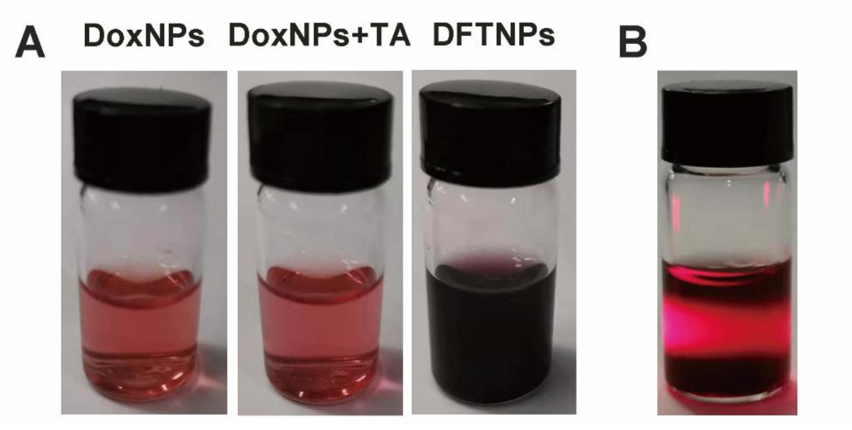

Figure S1. Observation on (a) DoxNPs, DoxNPs+TA and DFTNPs, respectively (pH=7.0). (b) Photograph of the Tyndall effect of DFTNFs suspension.

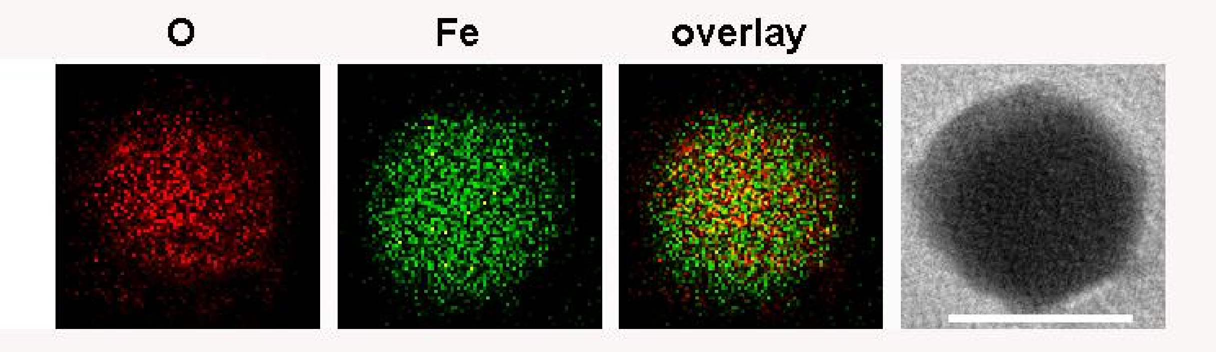

Figure S2. TEM image and the corresponding elemental mapping of Fe, O, and Fe+O combined. Scale bar: 100 nm.

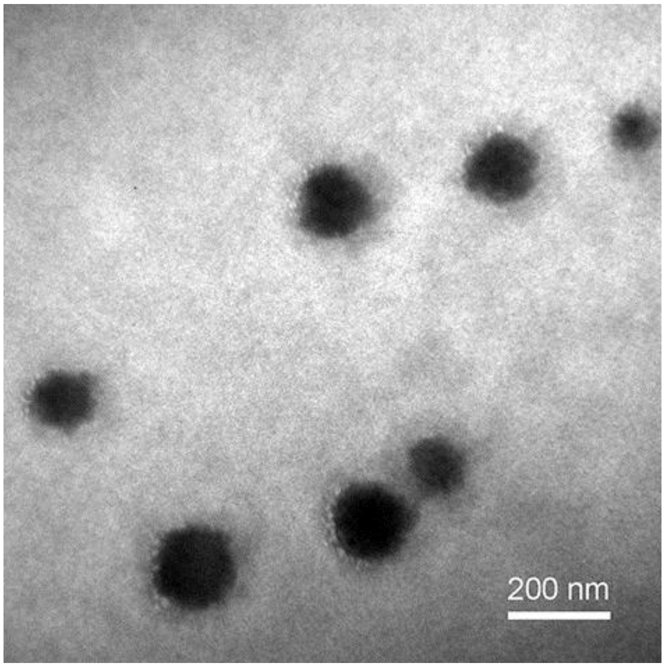

Figure S3. TEM image of DoxNPs.

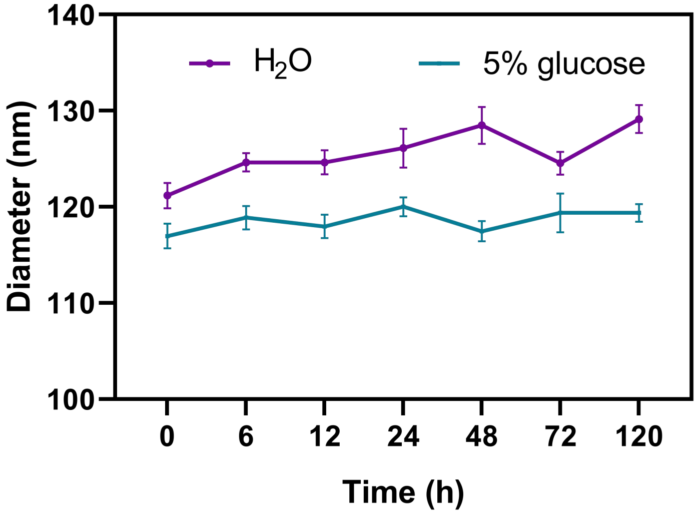

Figure S4. Size changes in 120 h observations in different buffer solutions.

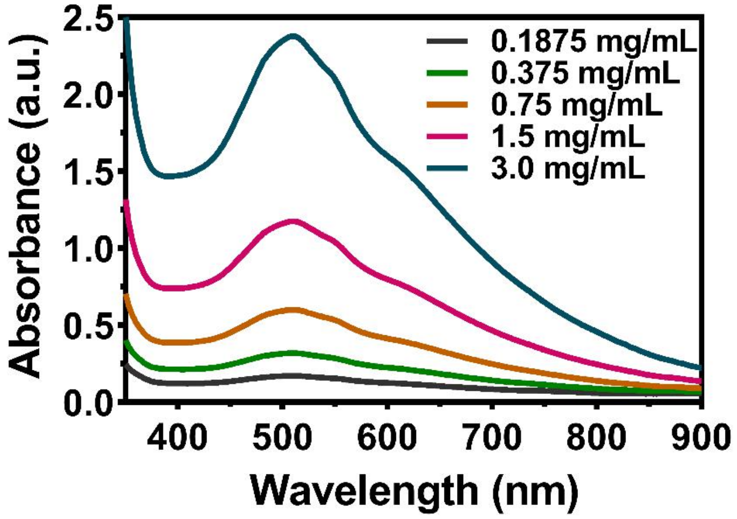

Figure S5. Absorption spectra of DFTNPs at different concentrations.

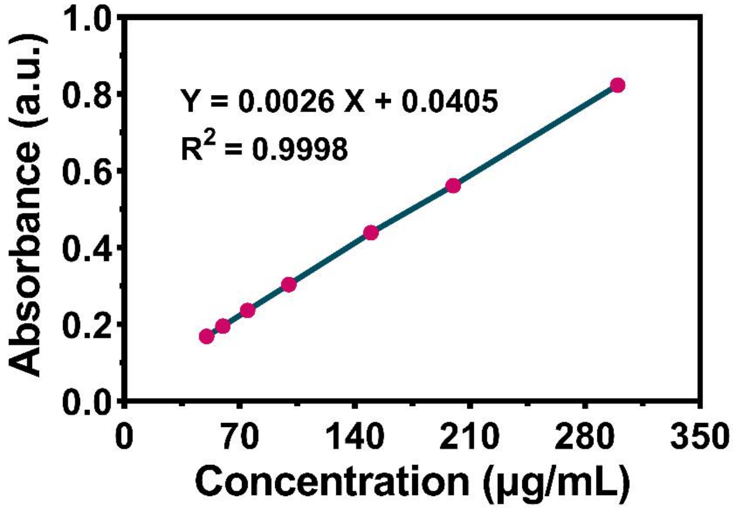

Figure S6. The standard curve of Dox constructed from UV-Vis-spectra measurements.

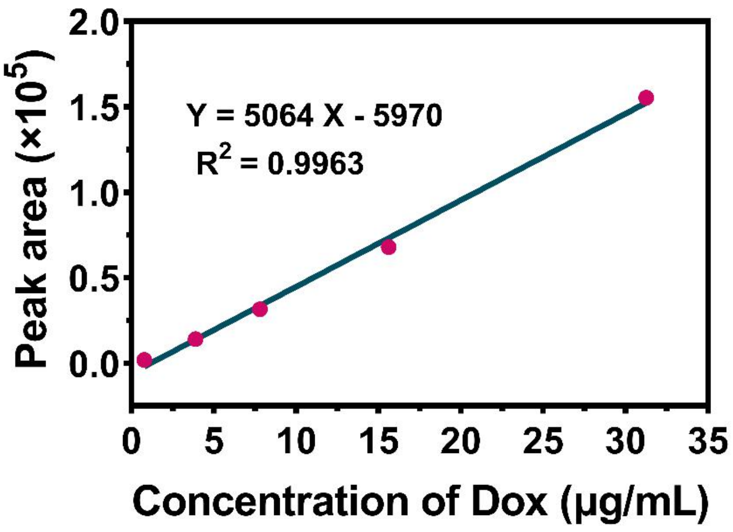

Figure S7. The standard curve of Dox constructed from HPLC measurements.

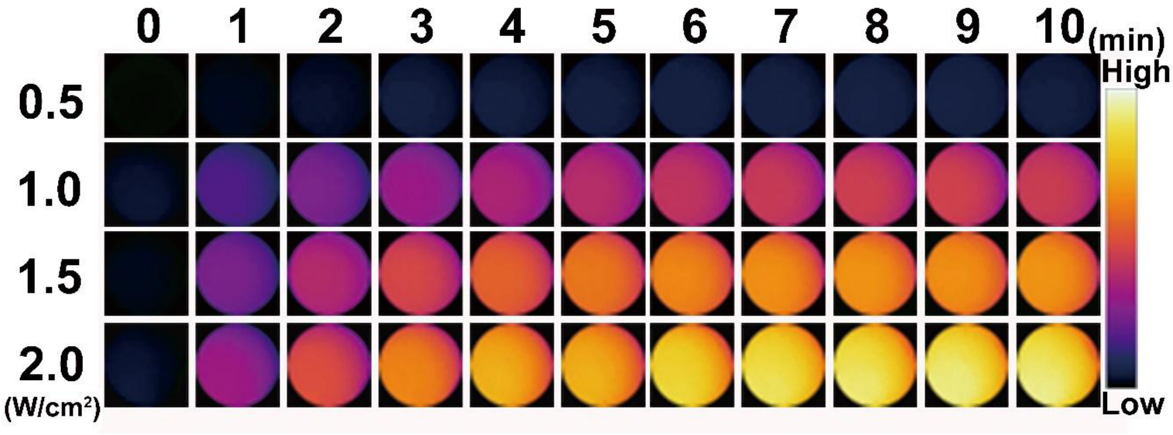

Figure S8. Photothermal images of DFTNPs dispersed in aqueous solution irradiated under increased power intensities (0.5, 1.0, 1.5, and 2.0 W/cm^2).

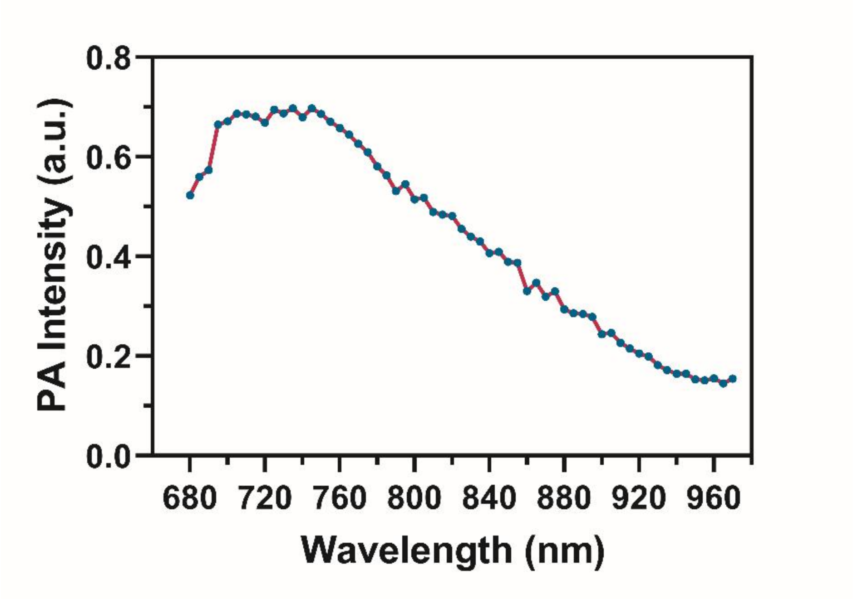

Figure S9. PA signal changes of DFTNPs by a laser irradiated at the wavelength range of 680-970 nm.

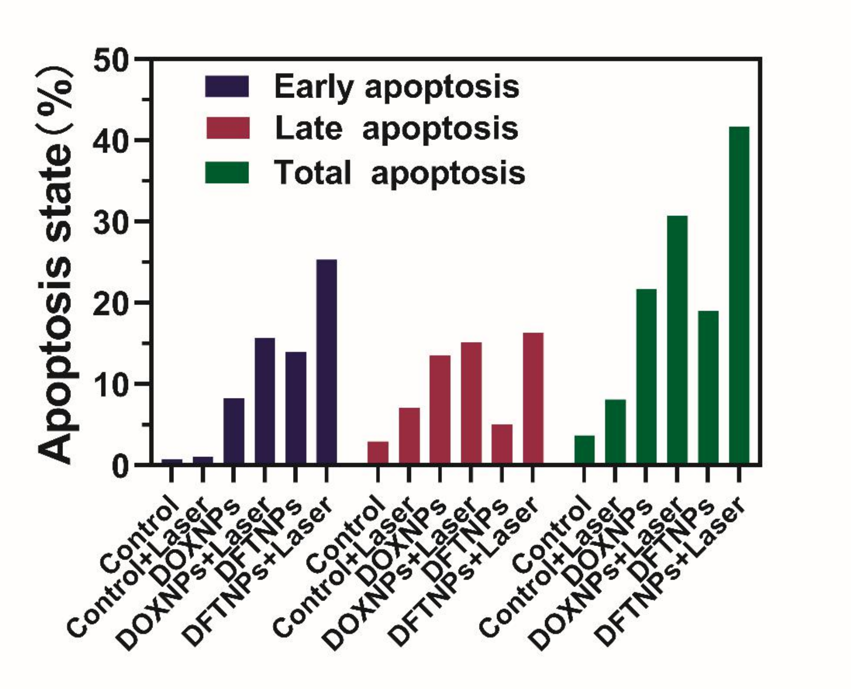

Figure S10. Quantitative analysis of cell apoptosis of MDA-MB-231 cells by flow-cytometry assay after various treatment.

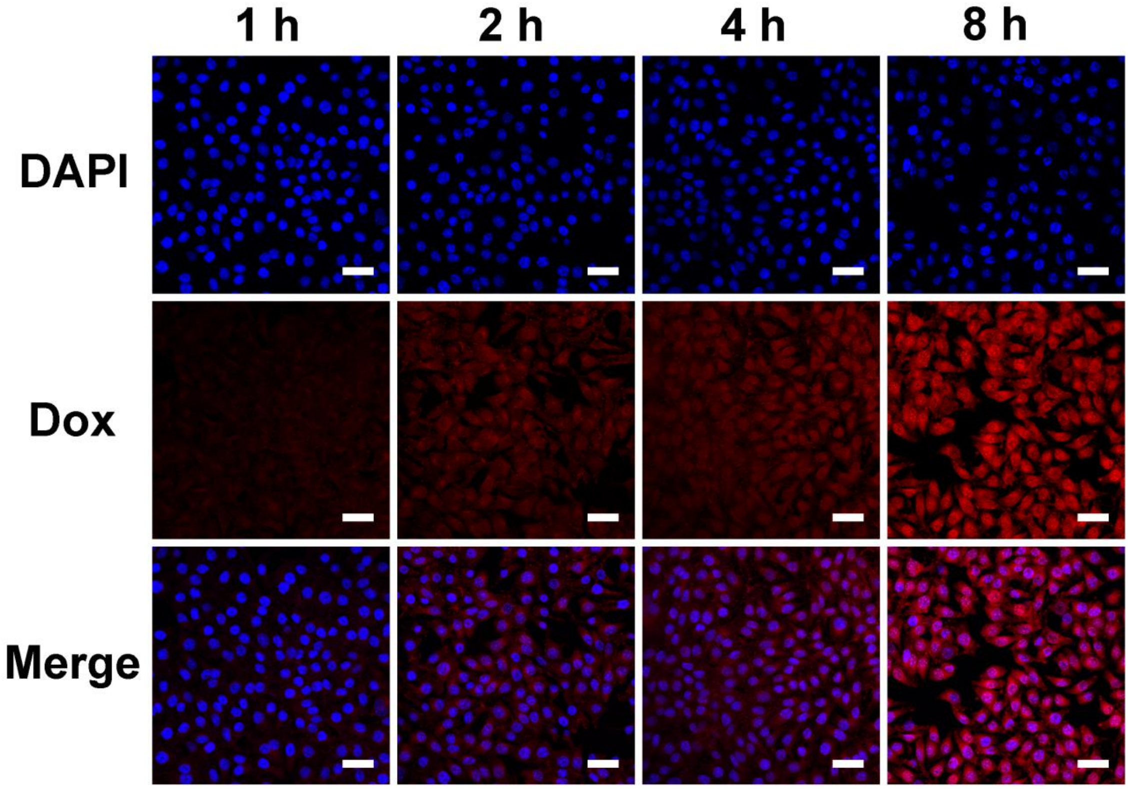

Figure S11. Confocal microscopy images of MDA-MB-231 cells co-incubation with DFTNPs after 1 h, 2 h, 4 h, and 8 h at a concentration of 40 μg/mL. The scale bar is 50 μm.

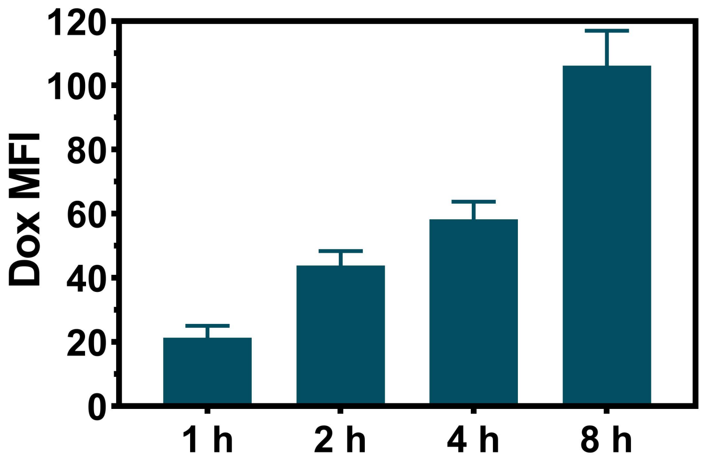

Figure S12. Quantification analysis of Dox fluorescence intensity in MDA-MB-231 cells after incubation for 1 h, 2 h, 4 h, and 8 h.

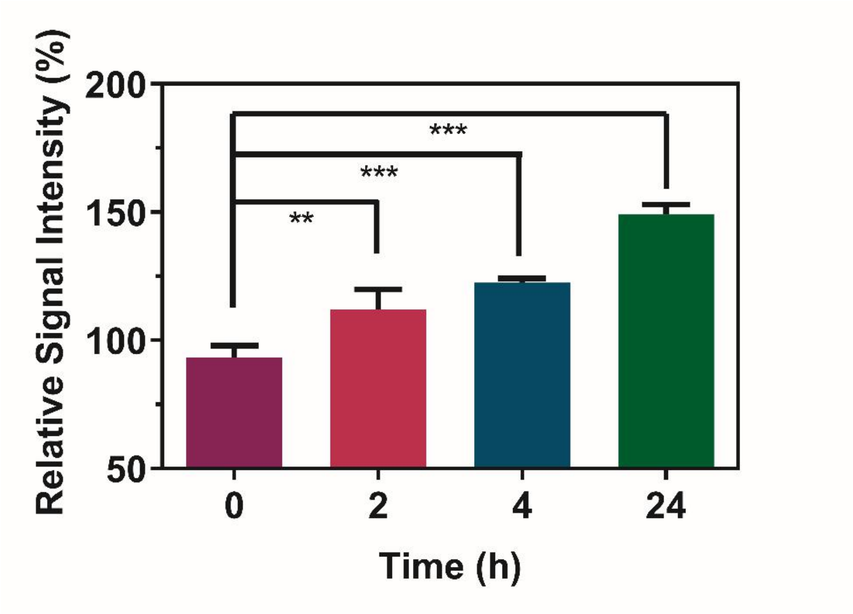

Figure S13. Corresponding relative T_1-weighted MRI signal intensity change between tumor region and nontumor region after intravenous administration of DFTNPs at certain time intervials.

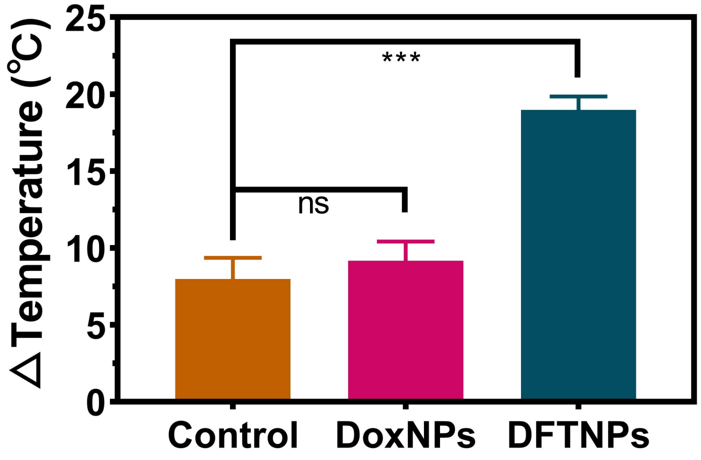

Figure S14. Corresponding tumor-site temperature elevation after laser irradiation.

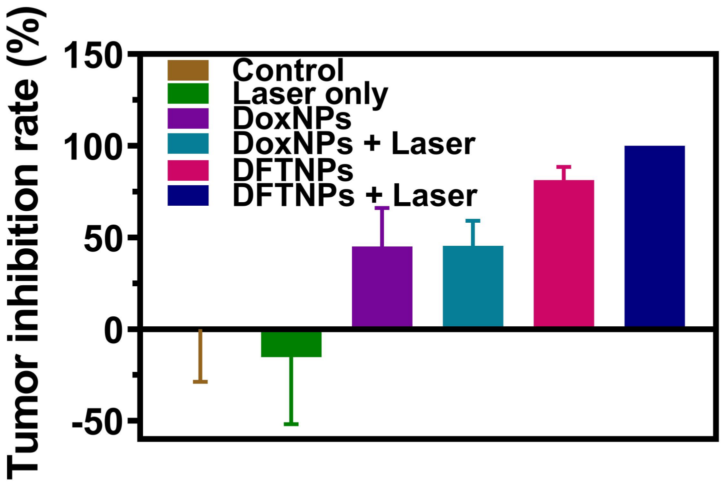

Figure S15. Tumor inhibition rate of six groups after various treatments.

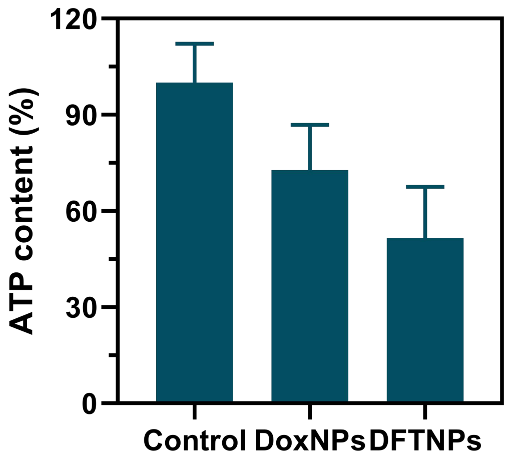

Figure S16. In vivo ATP content in different groups.

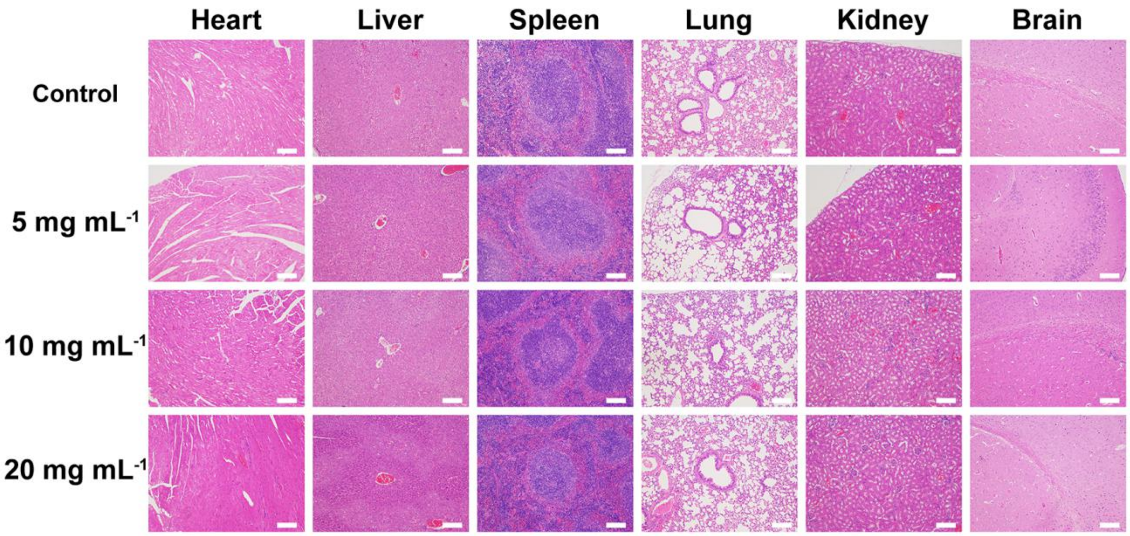

Figure S17. Histological secetions of heart, liver, spleen, lungs, kidneys, and brain from Kunming mice post-injection with different concentrations of DFTNPs at 30 d. The scale bar is 100 μm.
